# RF Sputtering of Gold Nanoparticles in Liquid and Direct Transfer to Nafion Membrane for PEM Water Electrolysis

**DOI:** 10.3390/membranes15040115

**Published:** 2025-04-07

**Authors:** Chandrakanth Reddy Chandraiahgari, Gloria Gottardi, Giorgio Speranza, Beatrice Muzzi, Domenico Dalessandro, Andrea Pedrielli, Victor Micheli, Ruben Bartali, Nadhira Bensaada Laidani, Matteo Testi

**Affiliations:** 1 Center for Sustainable Energy, Fondazione Bruno Kessler (FBK), 38123 Trento, Italy; ggottard@fbk.eu (G.G.); ddalessandro@fbk.eu (D.D.); micheli@fbk.eu (V.M.); bartali@fbk.eu (R.B.); laidani@fbk.eu (N.B.L.); testi@fbk.eu (M.T.); 2 Center for Sensors and Devices, Fondazione Bruno Kessler (FBK), 38123 Trento, Italy; speranza@fbk.eu (G.S.); pedrielli@fbk.eu (A.P.); 3 ICCOM, National Research Council (CNR), Sesto Fiorentino, 50019 Florence, Italy; beatrice.muzzi@unifi.it

**Keywords:** sputtering in liquids, gold nanoparticles, polyethylene glycol, nafion, catalyst-coated membrane, catalytic activity, water electrolysis

## Abstract

Sputtering onto liquids is rapidly gaining attention for the green and controlled dry synthesis of ultrapure catalysts nanomaterials. In this study, we present a clean and single-step method for the synthesis of gold nanoparticles directly in polyethylene glycol (PEG) liquid using radio frequency (RF) magnetron sputtering and by subsequently transferring them to Nafion ionomer, fabricating a catalyst-coated membrane (CCM), an essential component of the proton exchange membrane water electrolyzer (PEMWE). The samples were systematically characterized at different stages of process development. The innovative transfer process resulted in a monodispersed homogeneous distribution of catalyst particles inside CCM while retaining their nascent nanoscale topography. The chemical analysis confirmed the complete removal of the trapped PEG through the process optimization. The electrochemical catalytic activity of the optimized CCM was verified, and the hydrogen evolution reaction (HER) in acidic media appeared outstanding, a vital step in water electrolysis toward H_2_ production. Therefore, this first study highlights the advantages of RF sputtering in liquid for nanoparticle synthesis and its direct application in preparing CCM, paving the way for the development of innovative membrane preparation techniques for water electrolysis.

## 1. Introduction

Water electrolysis represents an attractive technology in the transition toward renewable and green energy systems amidst growing concerns about climate change and the need for sustainable energy solutions [1]. The primary advantage of water electrolysis lies in its ability to produce hydrogen, a versatile and clean fuel, without carbon emissions when powered by renewable electricity, making it an essential component of a future clean energy infrastructure [2]. Proton exchange membrane water electrolysis (PEMWE) is one such efficient and leading electrolysis technology that is witnessing great potential to provide large-scale hydrogen production [3]. The working mechanism of PEMWE and its core components are widely discussed in the literature [2,4]. It mainly consists of a proton exchange membrane that facilitates proton conduction, electrodes (anode and cathode) that enable the electrochemical oxygen and hydrogen evolution reactions, and CCM, which plays a dominant role in determining the overall performance and cost of the system [5]. The CCM is a composite layer where the catalyst, typically composed of precious platinum (Pt) group metals that ensure high performance and stability, is integrated with the membrane [4]. The commercially available Nafion, an ionomer membrane, has high proton conductivity and chemical stability and has become an essential component in PEMWE and similar technologies, where it serves as an all-in-one ion conductor and an efficient barrier layer toward active species [6,7,8,9]. Typically, CCMs are manufactured by tedious preparation processes followed by coating slurry ink mixtures of catalyst and ionomer [10,11]. This generally requires the production of large quantities of powdered catalysts, which are then mixed with ionomer solutions, often suffering from aggregation and coagulation that seriously affect the formation of the catalyst layer’s microstructure, performance, and overall cost [12,13,14]. Researchers are exploring various advanced strategies, such as using engineered metal catalysts and innovative preparation methods while improving efficiency and lowering material costs [11,15].

The sharp rise in demand for non-Pt catalysts has spurred research into developing alternative, cost-effective, scalable, and efficient catalysts for realizing the ambitious sustainable hydrogen landscapes. Gold nanoparticles (Au NPs) have emerged as one of the viable alternatives to Pt catalyst due to their advantageous unique chemical, physical, electronic, and electrocatalytic properties [16,17]. Au was considered a poor catalyst because the theoretical calculation showed a weak adsorption of hydrogen atoms on the Au catalyst surface [18]. Recent studies have demonstrated that gold nanoparticles, when appropriately synthesized and applied with atomic precision, can exhibit outstanding electrocatalytic activities comparable to benchmark metals while also offering the advantage of lower cost and increased stability under certain conditions [17,19,20,21]. This implies that the preparation method and substrate that affects the size tuning, morphology, and crystal surface helped to improve the functional electrocatalytic properties of nanosized Au [22,23,24]. For this reason, dry synthesis via sputtering can be advantageous over the traditional chemical synthesis of Au NPs, which typically involves multiple chemical reduction steps that are more prone to affect morphology, size, and colloidal stability, directly impacting the functional properties [25,26]. Moreover, a key challenge in integrating nanoparticles into electrolysis systems lies in their efficient synthesis and incorporation into catalytic membrane systems. Therefore, in light of improving future technologies, the green and ultra-pure preparation of catalysts through plasma deposition methods is being explored [27,28].

RF magnetron sputtering, a physical vapor deposition method, is a versatile technique that has recently extended its horizons onto liquid substrates, allowing the production of colloidal dispersions called nanofluids [28]. Sputtering in liquid media, in particular, presents a unique approach by enabling the deposition of nanoparticles in a solvent without the need for additional chemical reagents, thus offering a more environmentally friendly and scalable technique. It is a clean and single-step method that produces the monodispersed and ultrapure NPs without post-synthesis purification, and it is completely different from the wet synthesis that requires complex processing steps [27,29]. The NPs are composed of only the sputtered material, while the host liquid plays the role of the dispersion medium [30]. Recently, these nanofluids have found diverse applications such as in heat transfer, electronics cooling, biomedical, and space and defense [31]. The brief history of vacuum sputtering in liquids has yielded great achievements in the green production of various nanoparticles (NPs) [32]. A wide range of elements has been successfully developed in liquids with low vapor pressure, such as silicone oil, glycerol, ionic liquids, and polyethylene glycol [29,30,33]. Most of these studies reporting on the formation of nanoparticles in liquid via sputtering deal with the influence of deposition parameters. There are very few reports that demonstrate the utilization of such nanofluids realized through sputtering onto liquids, particularly for electrocatalysis applications [33,34]. This is mainly due to the difficulties involved in the efficient separation of the deposited nanomaterial from the liquid medium. Therefore, here, we report our innovative approach to prepare Au NPs by sputtering onto liquid and then utilizing the produced nanofluid for water electrolysis application.

This work focuses on the vacuum plasma synthesis of Au NPs directly in polyethylene glycol (PEG) as a liquid medium using the method of RF magnetron sputtering and duly characterized to confirm their size and morphology. Thereafter, the resulting nanofluid containing the Au NPs is directly transferred to the ionomer solution, without the need for trivial nanoparticle separation steps, and the CCM is thus fabricated. The CCM is then systematically investigated using FE-SEM, AFM, and XPS characterization techniques, confirming the transfer process. Furthermore, the CCM is post-treated, a simple yet effective optimization step that completely removed the PEG trapped in the membranes, which is probed using XPS chemical analysis. Finally, the HER electrochemical tests were performed on the optimized CCM that exhibited a superior performance in acidic media and compared with a commercial benchmark catalyst material, presenting a novel and feasible route to develop CCM via the sputtering technique in liquids, which is hereby discussed and demonstrated.

## 2. Materials and Methods

This paper focuses on the preparation of gold nanocatalysts through RF sputtering in a PEG liquid medium, resulting in a nanofluid and its characterization. In order to explore the electrocatalytic properties of the nascent gold nanoscale particles, a solution exchange has been performed to transfer the nanoparticles to the ionomer, targeted for fabricating a CCM. These membranes have highly homogeneous catalyst particle dispersion. The electrocatalytic activity of CCM was compared to those of a benchmark Pt ink prepared from commercial Pt-coated Vulcan carbon powder (Pt/C). The procedure for preparing the nanofluids and membranes is described as follows.

### 2.1. Materials

This study used commercially available polyethylene glycol (PEG) with a molecular weight of 600 g/mol (Merck KGaA, Darmstadt, Germany) as a low-vapor-pressure-liquid. A 2-inch gold metallic target (ACI alloys, 99.99% purity, San Jose, CA, USA) was used for sputtering. Nafion^®^, a perfluorosulfonic acid (PFSA)-based homogeneous alcohol-based solution with 5% dispersion (D5, Fuel Cell Store, Bryan, TX, USA), was used to prepare the membranes. Next, 20% Platinum on Vulcan carbon (Pt/C) (XC-72R, Fuel Cell Store, Bryan, USA) and 2-proponol (>99% purity, Merck, Milan, Italy) were used to prepare the benchmark ink. Sulfuric acid (ACS reagent, 95–98% purity, Merck, Milan, Italy) was used to prepare the 0.5 M electrolyte solution, and a glassy carbon (GC) electrode plate (1 mm thick, 5 × 5 cm^2^, type 1, Thermo Scientific, Milan, Italy) was purchased to use as an electrode support. Milli-Q de-ionized (DI) water with a resistivity of 18.2 MΩ-cm was produced in the laboratory.

### 2.2. Preparation of the Nanofluid

**Figure 1:**
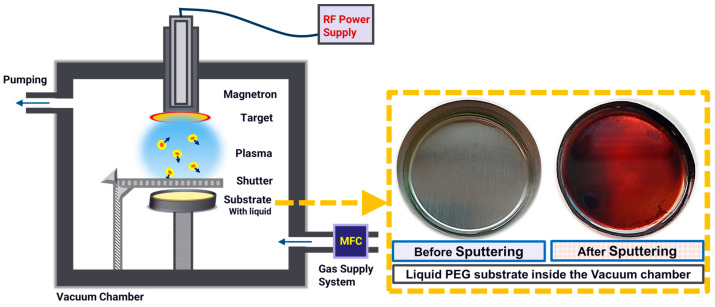
Schematic representation of the RF magnetron sputtering of Au into the PEG liquid. In the inset: vessels containing PEG liquid, before and after sputtering.

PEG is a physical solvent at room temperature with a viscosity of 170 cp, and it has a very low vapor pressure of <0.01 mbar, making it suitable for use as a liquid in high vacuum environments [35]. In an argon plasma, Au sputtering in PEG was performed using radio frequency (13.56 MHz) magnetron sputtering by mounting a 2-inch gold (Au) metallic target. Due to the hygroscopic nature of the PEG, it was pre-treated in a vacuum oven at 90 °C for 3 h, which removed all the volatile compounds, and a safe and controlled operation was ensured in the high-vacuum chamber. Next, 7 mL of pre-treated PEG was poured into a circular stainless vessel (10 cm in diameter) and spread uniformly over its surface. The vessel was then placed on the deposition stage inside the sputtering chamber. The schematic of the deposition chamber is shown below in Figure 1. The chamber was then pumped to an ultrahigh vacuum (UHV), to a base pressure of less than 2.8 × 10^−6^ mbar. Argon gas was then introduced at 21 sccm, bringing down the chamber operating pressure to 1.5 × 10^−2^ mbar. A pre-sputtering was performed for 10 min to clean the target. The depositions were performed at a plasma power of 50 W (V_bias_ 170 V) (CESAR RF Power Generator, Advanced Energy, Wilmington, NC, USA) for 2 min. A photograph in Figure 1 (inset) also shows that the colorless PEG has turned into a uniform color upon Au sputtering.

### 2.3. Fabrication of the Membrane

The catalyst-coated membrane (CCM) was fabricated by mixing 2 mL of the as-prepared nanofluids of PEG containing Au NPs with 1.5 mL of D.I. water, 3.5 mL of 2-propanol, and 100 µL of ionomer solution (Nafion^®^, D5), taken into a 15 mL centrifuge tube and placed on a vibrator (Model: F20220176, VELP SCIENTIFICA, Usmate, Italy) at 40 Hz for at least 5 min for vigorous mixing. After mixing, the tube with the solution was placed in a bath ultrasonicator (TRANSSONIC T 460/H, Model: D-78224, Singen, Germany) for 30 min for improved dispersion. Afterward, the solution was poured into a Petri dish, which was then placed on a hot plate magnetic stirrer (Model: SP88857205, Isotemp, Fischerbrand, Chelmsford, MA, USA) and heated to 90 °C for 3 h to generate a soft membrane with a homogeneous and uniformly pink color. The transparent PEG was removed by introducing fresh DI water followed by gentle shaking for 30 sec. Finally, the film dried in the vacuum oven (Model: DZF-6020, MTI Corp., Richmond, VA, USA) at 70 °C for 2 h, resulting in an uncracked membrane. A post-treatment of the CCM was also carried out, as an additional optimization step, to completely remove any residual trapped PEG chains surrounding the Au NPs. Such an optimization step consisted of washing the membrane by dipping it in hot D.I. water at 90 °C for 15 min and then sonicating it in an ultrasonic bath for 5 min. The washing step was repeated twice, then the membrane was dried in a vacuum oven at 70 °C for 2 h. A pure reference membrane composed of only the ionomer, without Au NPs, was prepared in a similar process except for the post-treatment process, which was not necessary as PEG was not present in the formulation.

### 2.4. Preparation of the Pt/C Ink

The electrocatalytic properties of Au NPs were compared to those of the commercial benchmark Pt/C catalyst material. The ink was prepared starting from standard recipes found in the literature [36,37]. Briefly, 3 mg of Pt/C powder was dispersed in 1.5 mL of Milli-Q DI water, 3.5 mL of 2-propanol, and 100 µL of PFSA. The suspension was mixed in an ultrasonic bath for 30 min. Finally, 350 µL of ink was dropped on a GC electrode, followed by drying at 50 °C on a hot plate.

### 2.5. Characterization Methods

#### 2.5.1. Nanofluid

A scanning transmission electron microscope (STEM, Thermo Scientific Talos F200X, Milan, Italy) equipped with a Ceta 16 M camera, using a single tilt sample holder with a high-angle annular dark-field (HAADF) at an acceleration voltage of 200 kV, was employed to observe morphology at high magnification. An OmegaScope AFM (Horiba Jobin Yvon, Paris, France) with an HQ:NSC14/Al BS (a force constant of 5 N/m, a frequency of 160 kHz) tip and an amplitude of 20 nm was used. The samples for STEM and AFM were prepared by diluting the nanofluid in deionized water in 1:20 and drop-cast onto the respective substrates (Cu metal grid coated with a carbon film for STEM and a Si substrate for AFM) and allowing them to dry at room temperature. UV–vis spectra of the nanofluid were monitored on a spectrophotometer (Jasco V-670, Cremella, Italy) operated in the 200–800 nm range at a resolution of 2 nm in absorption mode. The analysis was performed on the samples diluted with D.I. water (1:20). Next, 4 mL of the diluted sample was then taken in a quartz cuvette to measure against the pure PEG diluted in a D.I. 1:20 ratio. The X-ray diffraction (XRD) analyses were performed using an X-ray diffractometer (Italstructures, model APD 2000, Trento, Italy) with Cu Kα radiation (40 kV and 20 mA). The crystallite size of Au was calculated using the Scherrer equation. The sample for XRD was prepared by diluting 2 mL of nanofluid in D.I. water (1:20) and performing the ultracentrifugation at 3220 g for 60 min. The sediment was dropped onto a Si substrate, followed by a baking step that removed the water. The field emission scanning electron microscopy (FESEM, Thermo Fisher, Milan, Italy) equipped with energy-dispersive X-ray spectroscopy (EDX) was also used to further characterize Au NPs deposited on the Si substrate.

#### 2.5.2. Membrane

The CCM membrane was characterized using XPS, FE-SEM, and AFM to analyze the chemical compositions and particle distribution subjected to optimization strategies. X-ray photoelectron spectroscopy (XPS) was used to examine the changes in the surface functional groups and identify the surface composition of the produced samples, using an X-ray Photoelectron Spectrometer Axis DLD Ultra from Kratos—Manchester, UK. The wide spectra were acquired at 160 eV pass energy, while the high-resolution spectra were acquired at 20 eV pass energy, leading to a resolution of ~0.3 eV. The XPS data were analyzed using homemade software (RxpsG version 3.2) based on the R platform [38]. The energy reference of the binding energy (B.E.) scale was the Fermi level measured on a sputter-cleaned Ag reference. Charge compensation was applied to acquire the spectra. Optimal analysis conditions were found by reducing the FWHM of the spectrum and increasing their intensities. B.E. alignment was performed by placing the lower C 1s components at 285 eV for the Au-Nafion as-prepared sample and at 284.4 eV for the pure and post-treated samples. After a Shirley-type background subtraction, the spectra were fitted with a Gaussian peak line shape. FE-SEM, AFM, and XRD were also used to analyze the surface morphology, topography, and crystallinity of the CCM with no additional sample preparation.

### 2.6. Electrochemical Evaluation

**Figure 2:**
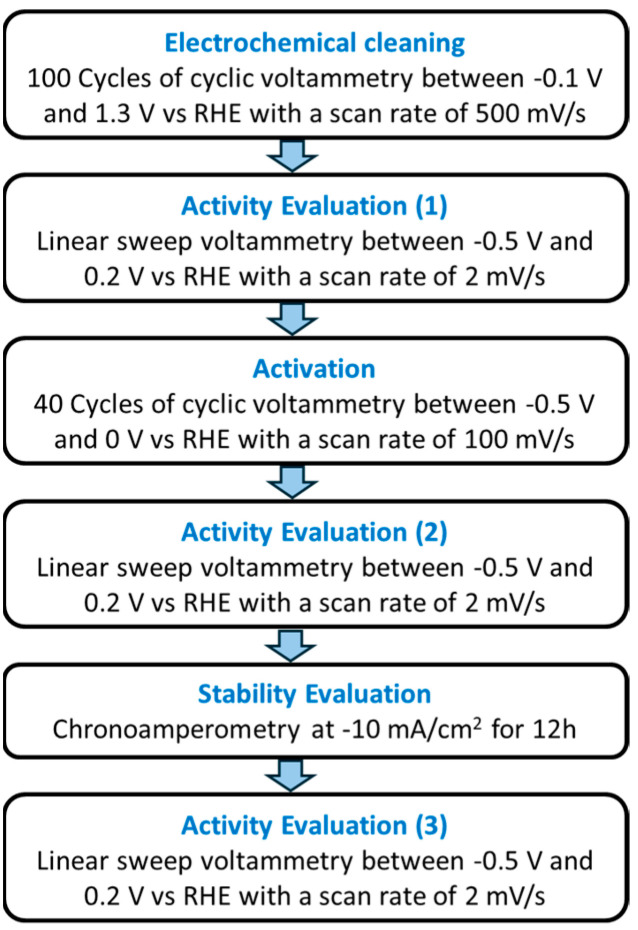
Schematic representation of the electrochemical test protocol used for the analysis of the catalytic activity and stability of CCM.

Electrochemical tests were performed on the CCM membrane to evaluate the HER water electrocatalysis in an acidic media using a three-electrode cell (Gaskatel FlexCell^®^, Kassel, Germany) connected to an SP-150 Biologic potentiostat (BioLogic GmbH, Gottingen, Germany). A HydroFlex^®^ RHE electrode (BioLogic GmbH, Gottingen, Germany) was employed as the reference electrode. The FlexCell was filled with 0.5 M of H_2_SO4 electrolyte solution to simulate the acidic environment of PEM electrolyzers. The CCM sample was properly mounted on a glassy carbon (GC) electrode, and the working electrode’s area exposed to the solution was 3 cm^2^. Starting from the methodologies and techniques most used in literature [39,40], a testing protocol at room temperature was established to evaluate the catalytic activity and stability of Au NPs, including an initial phase of electrochemical cleaning and activation, a second phase to assess catalytic activity using linear sweep voltammetry (LSV), and a third phase to evaluate stability through chronopotentiometry (CP). The detailed test protocol using the three-electrode cell offers accurate and reproducible screening of electrocatalytic activity close to those obtained in real PEM electrolyzers and eliminates the usage of the expensive apparatus, as described in Figure 2. Prior to electrochemical cleaning, a stabilization phase and iR drop calculation were performed. Stabilization occurred at an open-circuit voltage (OCV) for 20 min while purging the solution with nitrogen. The iR drop was determined using electrochemical impedance spectroscopy (EIS). To assess the effectiveness of the activation process, catalytic activity evaluations were performed via LSV before and after activation. An additional activity check via LSV was conducted after 12 h of CP to better evaluate catalyst stability. The test set-up and protocol were duly validated using the benchmark Pt/C material.

## 3. Results and Discussion

### 3.1. Nanofluid Analysis

**Figure 3:**
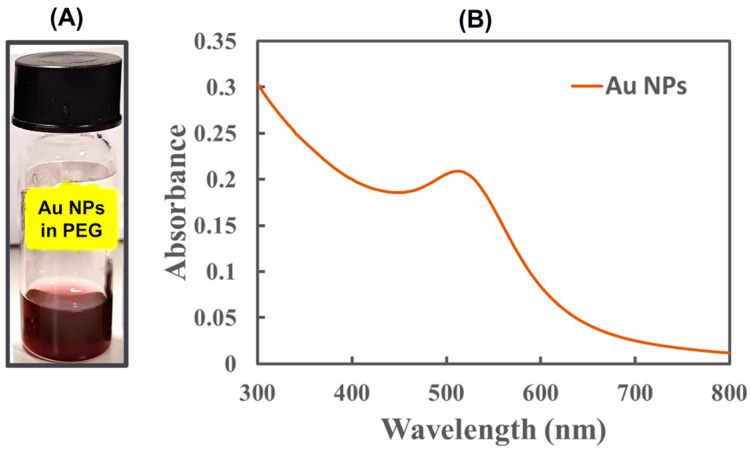
(**A**) Photograph and (**B**) UV–vis absorption spectrum of the PEG nanofluid with Au NPs.

A nanofluid containing nanoscale gold particles (Au NPs) was prepared using RF magnetron sputtering into liquid PEG, following the optimized deposition process protocol described in Section 2.2. The uniform and very homogeneous appearance of the nanofluids indicates the formation of monodispersed and stable nanocatalysts. The nanofluid had a uniformly dark pink color, as shown in Figure 3A, owing to the Au size-dependent surface plasmon resonance (SPR), a phenomenon that occurs due to the collective oscillation of conduction electrons induced by an incidental electromagnetic beam. Characteristic peaks of SPR vary, depending upon the core diameter of the Au NPs and the surrounding media. Figure 3B shows the UV–visible absorption spectrum that quickly identified the formation of the gold nanoparticles, with its SPR peak positioning at 520 nm. The spectrum showed a similar absorption peak pattern for Au NPs that had a sub-nm size range reported in the literature [23,24,26]. Moreover, these nanofluids were also found to have a long shelf life of several months and remained homogeneous.

**Figure 4:**
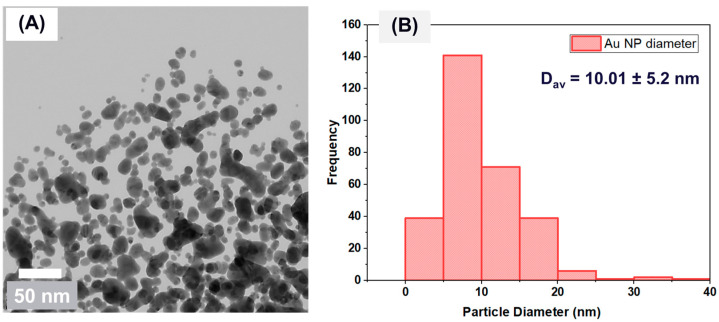
(**A**) STEM image acquired on nanofluid containing Au NPs and (**B**) a histogram of the numerical size distribution of the Au NPs’ diameters.

STEM and AFM techniques were used to characterize the shape and dimensions of the deposited NPs. We utilized STEM with a high-angle annular dark-field (HAADF), a powerful technique to probe structural morphologies at a sub-nm scale, to analyze the size and morphology of the colloidal nanoparticles. The STEM image shown in Figure 4A proves that many nano-scale particles with spherical morphology were formed through RF sputtering in the PEG medium and were polydispersed. Figure 4B shows the numerical size distribution histogram of the Au NPs, indicating that the average diameter of the Au NPs is 10 nm with a standard deviation of ±5.2 nm. Moreover, the high-magnification image demonstrates that the nanoparticles showed a tendency to come in proximity to each other.

**Figure 5:**
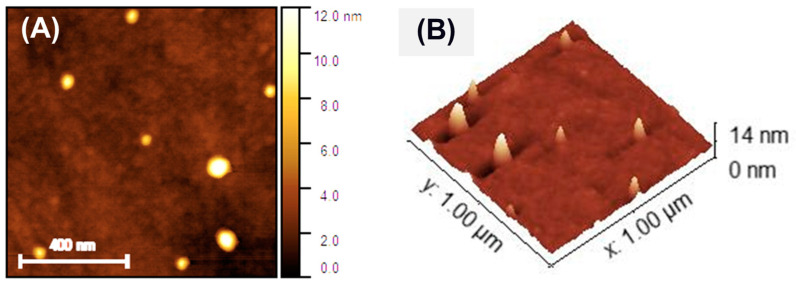
(**A**) AFM topographical map and (**B**) 3D view of the map of the Au NPs.

AFM topographical analysis was carried out on an area of 1 µm × 1 µm, as shown in Figure 5. The apparent lateral diameter of the Au NPs is in the range of 28–80 nm, as shown in Figure 5A. However, a measurement of the NPs dimension should rather rely on the vertical height, as the lateral dimension is the result of the convolution of the nanoparticle dimension and the tip shape (<8 nm). The vertical dimension of the NPs in Figure 5B is in the range of 5–12 nm, which agrees with the dimension derived from STEM analysis. The absence of features such as lobes is also in agreement with the STEM analysis result, which describes the NPs as having a spherical morphology and being monodispersed in the PEG medium. It can be noticed that the formed nanoparticles may have a very thin outer layer, which may be due to the presence of molecular PEG chains attached due to the PEG growth medium.

**Figure 6:**
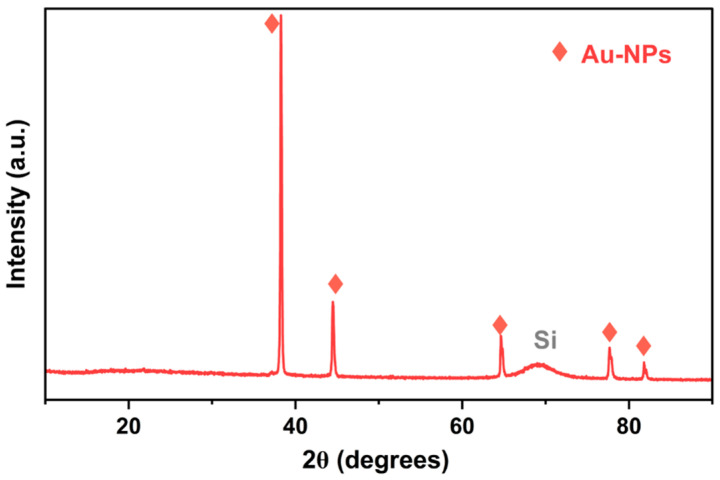
XRD crystalline structural analysis of the Au NPs dried on a silicon substrate.

XRD was performed to confirm the purity and crystalline phase of the sputtered nanoparticles suspended in the nanofluid. Since XRD analysis cannot be performed directly on nanofluids, a small quantity of dried NPs was obtained using a separation process through centrifugation (~0.5 mg for 2 mL of nanofluid), as described in Section 2.5.1. Figure 6 shows the XRD pattern acquired on the Au NPs dried on a silicon substrate. The characteristic peaks of the Au NPs identified 2θ values of 38.2°, 44.3°, 64.6°, 77.6°, and 81.7°, which correspond to the (111), (200), (220), (311), and (320) planes, indicating the face-centered cubic (FCC) crystalline structure of Au [24]. A broad peak is also visible around 70°, attributable to the signal coming from the Si substrate. The crystallite size of the Au NPs calculated using the Scherrer equation was 71 nm, larger than the real particle size as measured using STEM. To understand this discrepancy in crystallite size, we performed an FE-SEM investigation on the collected powder on a silicon substrate.

**Figure 7:**
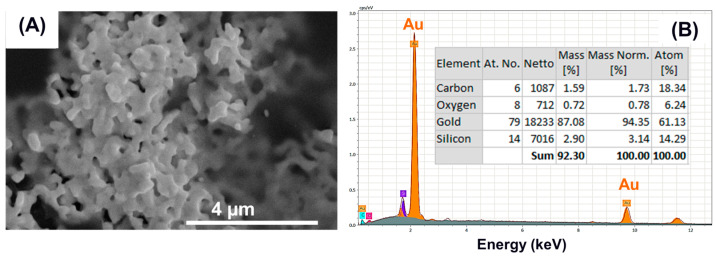
(**A**) FE-SEM image and (**B**) EDX spectrum of the Au NPs collected onto a Si substrate.

The FE-SEM analysis results shown in Figure 7A show the morphology of the collected Au NPs powders through separation using centrifugation. The nanoparticles show a flower-petal-like morphology with a size range of 0.4–2.5 µm. This is a clear change in morphology and size as compared to the STEM analysis performed on the as-prepared particles without undergoing the separation process. Figure 7B shows that the EDX elemental analysis confirmed that the nanoparticles are composed of pure gold with fewer carbon and oxygen contaminations, most probably originating from PEG residues. The separation process strongly affected the morphology and size of the particles while maintaining the chemical purity of the sputtered gold. Therefore, it can be hypothesized that nascent particles retain their monodispersed state while surrounded by the growth medium wherein they have nucleated. However, as the surrounding PEG chains are removed following the separation process, particle coalescence occurs due to the strong interaction between the pure metallic gold surfaces that form aggregates. A similar observation was made for gold nanoparticles synthesized through the chemical solution method using ligands [23]. This observation indicates that the separation of Au NPs from the growth medium may not be an ideal choice if we aim to exploit the promising features of the nascent particles. Hence, we developed the direct transfer method of sputtered Au NPs in a PEG medium to the ionomer membrane subjected to optimization.

### 3.2. Nanomembrane Analysis

**Figure 8:**
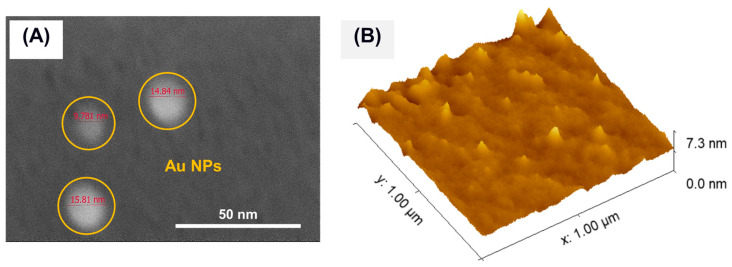
(**A**) FE-SEM high-mag image and (**B**) AFM surface topography of the Au NPs loaded on the ionomer membrane (CCM).

The ionomer membrane loaded with gold nanoparticles was prepared to retain the nascent properties of the nanosized gold produced using the sputtering technique. The CCM membrane had a thickness of 1.8 µm and a root mean square surface roughness of 75–110 nm, as measured with a surface profilometer (KLA Tencor). Its surface morphology was investigated using FE-SEM (without any metal layer coating) and AFM in semi-contact mode. Figure 8A,B show the FE-SEM high-magnification image and AFM surface topography (acquired on an area of 1 µm × 1 µm), respectively. The analyses confirm the presence of gold nanoparticles, homogeneously distributed and embedded throughout the ionomer homogeneous phase with a strong surface interaction. As can be seen in the high-magnification FE-SEM image in Figure 8A, the nanoparticles are spherical in morphology, monodispersed, and show a size range of 9–15 nm. This means that Au NPs have been successfully transferred to the ionomer membrane in their nascent state without undergoing any coalescence or aggregation. The transfer process was further confirmed with XRD analysis directly performed on the CCM. The XRD identified the 2θ peak at 38.2°, which corresponds to the characteristic (111) planes of Au, confirming the presence of Au NPs in the polymer matrix. Moreover, the crystallite size calculated using the Scherrer equation was 14 nm, which closely matches the value obtained using STEM analysis. This means that NPs are in their monodispersed state but not as aggregates. This result is a further confirmation that the transfer process of nanoparticles from the nanofluid to the membrane was effective, even in maintaining the nanoparticles in their original condition, i.e., monodispersed in the host matrix.

**Figure 9:**
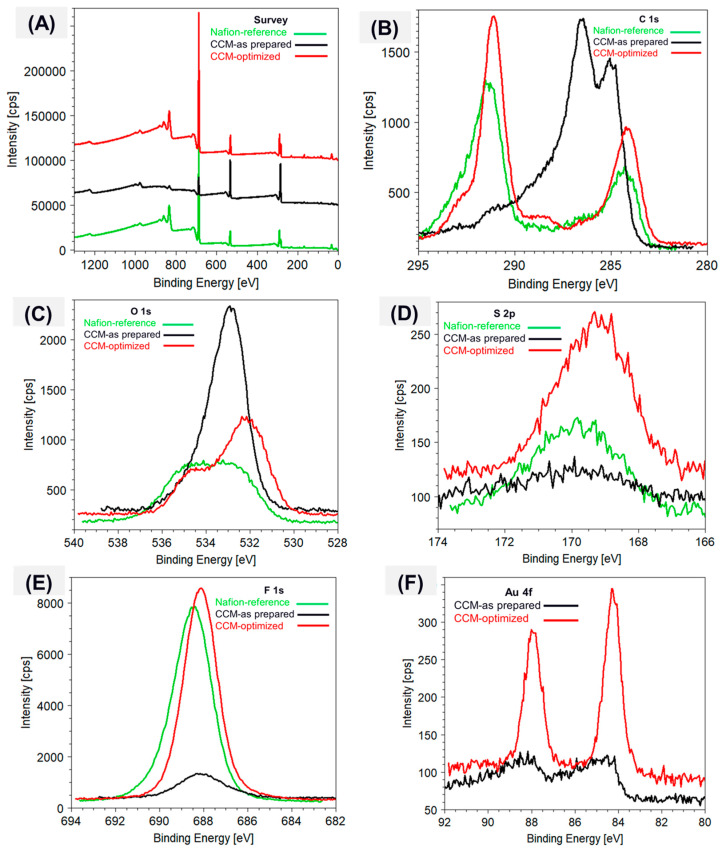
High-resolution XPS spectra acquired from the pure membrane (Nafion reference, green line), Au NP-loaded membrane (CCM-as prepared, black line) and the optimized membrane (CCM-optimized, red line): (**A**) survey, (**B**) C 1s, (**C**) O 1s, (**D**) S 2p, (**E**) F 1s, and (**F**) Au 4f core lines.

The optimization of the CCM preparation process to completely remove any PEG residuals was performed with the support of XPS analyses. This technique allows us to identify the surface elemental composition and chemical nature of the analyzed material. Therefore, using XPS, we analyzed the as-prepared CCM membrane loaded with Au NPs (later identified as “CCM-as prepared”), the post-treated CCM membrane (later identified as “CCM-optimized”), and, finally, the pure Nafion membrane without any Au NPs, which served as a reference (later identified as “Nafion-reference”). The XPS spectra and the core lines of the different elements acquired on the three samples are shown and compared in Figure 9. The survey spectrum in Figure 9A shows only the identified constituent elements, with their respective binding energies (B.E.), carbon (C 1s), oxygen (O 1s), sulphur (S 2p), fluorine (F 1s), and gold (Au 4f) representing the chemical purity of the prepared samples.

The C1s core line of the pure Nafion membrane (Figure 9B, green line) shows the typical main component at ~292.53 eV, expected for carbon in the -CF_2_- backbone configuration, and a secondary component at 284.40 eV due to adventitious carbon. As for oxygen, it is present in two different configurations within the chemical structure of Nafion, giving rise to a broad O 1s peak (Figure 9C, green line) composed by two components: the one at higher B.E. (~534.93 eV) correlates to oxygen present in the ether groups of the side chains (–F_2_C–O–CF_2_–) and that at lower B.E. (~532.77 eV) to oxygen present in the sulfonate groups (–SO_3_−). Both the S 2p spectra and F 1s (Figure 9D,E), green line) show, on the contrary, a single peak at ∼169.83 eV and ∼688.38 eV, respectively. All of the above-recognized binding energies are in good agreement with the literature [41].

Looking at the C 1s core line of the as-prepared CCM membrane (Figure 9B, black line), a totally different chemical structure is evident from the XPS analysis. The C 1s peak is dominated in this case by the signal of C-H and C-OH bonds at 285 eV and ~286.54 eV, respectively, while that of pure Nafion assigned to the C-F bonds, typically at B.E. ~291 eV, is rather weak. Correspondingly, the O 1s peak (Figure 9C, black line) shows a dominant component at 532.92 eV, attributable to C-OH bonds, while the S 2p peak at ~169.92 eV and that of F 1s at ~688.27 eV displays a clear reduction in intensity. At the same time, a doublet is visible at ~84.5 eV and ~88.5 eV, which can be assigned to the 4f_7/2_ and 4f_5/2_ levels of Au, even if the signal is rather weak. These results confirm that Au NPs have been successfully incorporated into the Nafion membrane but also highlight that the transfer process leaves a significant amount of PEG residue on the membrane surface, which masks the underlying membrane [42].

Interestingly, the surface chemistry of the optimized CCM membrane (Figure 9, red line) strongly differs from that of the as-prepared CCM, rather resembling that of the pure Nafion. We can observe from the C1s core line (Figure 9B, red line) that the peak is characterized by the same two intense components at ~292 eV and ~284 eV, previously identified as those typical of Nafion; moreover, the signals, due to the C-H and C-OH groups, are attributable to PEG residues that almost disappeared. Such a result can be well justified with the efficient removal of PEG residues due to the post-treatment step of the CCM, which leaves a cleaner Nafion-like surface. This is also confirmed by the O 1s, S 2p, and F 1s core line shapes and intensities (Figure 9C–E), red line), which better match those obtained from the reference sample [43]. At the same time, the intensity of the Au 4f doublet (Figure 9F) appears much stronger in the optimized CCM sample (red line) with respect to the as-prepared one (black line), indicating once more that the post-treatment was effective in removing PEG, thus allowing not only the Nafion membrane but also the embedded Au NPs to be better exposed on the surface [17,19,42]. It should also be noted that F 1s, S 2p, and Au 4f in the post-treated sample (CCM-optimized) shift to slightly lower binding energies compared with the same signature reported for the pure and as-prepared membranes. This shift can be attributed to the negative charge variations on the surface structure of the Au NPs due to the influence of the capped PEG that eventually modulated the stronger preferential bonding with the S-donors of the Nafion structure, thus forming Au-S bonds [17,41]. Moreover, the low B.E. differences (less than 0.5 eV) among the F 1s, S 2p, and Au 4f indicate improved charge transfer interplay due to the intimate interaction with the metallic gold nanoparticles [19,41].

All the above findings confirm the crucial role played by the simple post-treatment step that effectively removed the trapped PEG and improved the interaction of Au NPs with the ionomer. Therefore, it can be concluded that the XPS patterns verify the successful transfer of the sputtered gold nanoparticles to the ionomer membrane with a stronger surface interaction.

### 3.3. HER Catalytic Activity

**Figure 10:**
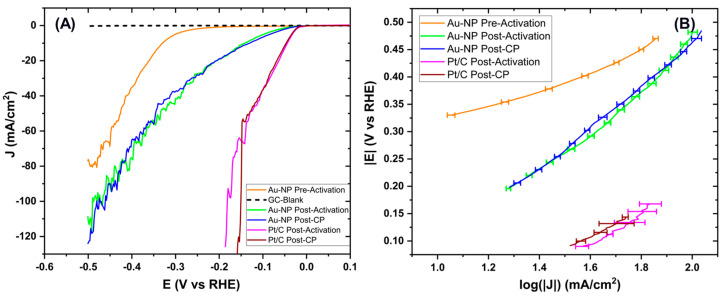
(**A**) LSVs of the sample supported on GC performed before and after activation and at the end of CP. (**B**) Onset potential and Tafel slopes of the LSVs measured.

Electrochemical tests conducted in acidic H_2_SO_4_ electrolyte solution evaluated the HER catalytic activity of the optimized CCM sample against the benchmark Pt/C. Figure 10A shows the measured linear sweep voltammetry (LSV)s, showing the hydrogen evolution reaction of the sample. It can be observed that all curves exhibit noise, especially at higher potentials (around −0.5 V vs. RHE for Au NPs and around −0.15 V vs. RHE for Pt/C), due to bubble formation during hydrogen evolution. The effect of the bubbles was mitigated by filtering the signal using a 29th-order moving average. The root mean square errors (RMSEs) for the signals before activation, after activation, and after chronopotentiometry (CP) are 1.0, 1.8, and 1.9 mA/cm^2^, respectively. It can be noted from the LSVs that, prior to activation (orange curve), hydrogen is being produced at the higher onset potential (−0.29 V vs. RHE), whereas the post-activation (green curve), achieved through 40 CV cycles, improved the performance as evidenced by a decrease in onset potential (−0.05 V vs. RHE) and an increase in current density values. This indicates more efficient hydrogen production subjected to post-activation. The post-CP (blue curve) measured after the 12-h CP stability test resembles the post-activation LSV (green curve) and is within the error range. Regarding the Pt/C curves, the measurements taken after activation and after the stability test (CP) have been reported. It is noted that the curves are very similar; indeed, there is a slight improvement in activity at higher potentials (over 0.15 V vs. RHE) after the stability test.

Figure 10B displays the Tafel slopes calculated for each curve starting from the onset potential. The onset potential and Tafel slope values obtained from the fitted LSV signals are reported in Table 1. For the Au NPs, the Tafel slope values tend to increase after activation, stabilizing at around 38 mV/dec before and after the stability test. A lower Tafel slope (around 20 to 30 mV/dec) and lower onsets (−0.02 V vs. RHE) are observed for benchmark Pt/C as reported in the literature [44]. This indicates that the rate-limiting step of the reaction is the Heyrovsky step (Au-H + H⁺ + e^−^ ⟶ Au + H_2_), governed by electrochemical desorption. This conclusion is reasonable, as the analyzed electrode shows a noisy signal caused by hydrogen bubble formation and detachment from the surface. This demonstrates that CCM produces hydrogen and exhibits high stability over time.

**Figure 11:**
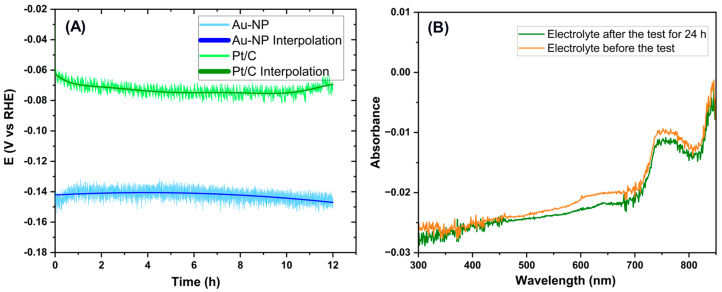
(**A**) Stability test via CP measured at 10 mA/cm^2^ for 12 h showing along with the interpolation result applied on the CP signal. (**B**) UV–vis absorption spectra of the electrolyte before and after the HER test.

To observe the slight performance degradation, the voltage variation was measured from the CP curve (Figure 11A), obtained by setting a current density of 10 mA/cm^2^, representing the electrode’s degradation over time. The bubble effects are visible even during the CP stability test and create noise in the potential signal. Observing Figure 11A, a slight decrease in the signal can be seen during the initial phase of the test (up to about one hour), likely due to double-layer stabilization. After the first hour, the signal remains almost stable, with a slight visible degradation. To quantify the degradation, the signal was interpolated using a third-order polynomial interpolation ($y(x)=\sum_{i=0}^{3}a_{i}x^{i}$). The red curve in Figure 11A shows the interpolation results, and the calculated RMSE was 4.3 × 10⁻⁹ V. The interpolation is not highly accurate for the potential signal during the initial hours of the test (up to about 2 h). However, this is negligible as no degradation effects are present during this phase. The final phase of the test is well-interpolated, allowing for the calculation of the degradation coefficient, which was found to be—(0.57 ± 0.08) × 10^−3^ V vs. RHE/h, corresponding to—(570 ± 80) mV/1000 h. Moreover, we have also tested the electrolyte solution using UV–vis spectroscopy, wherein the CCM was immersed over 24 h. The spectra recorded before and after the electrochemical tests were carried out remain unaffected, as shown in Figure 11B. The negative absorption of the spectra is only due to the very dilute and emissive nature of the electrolyte. Neither the release of any residue nor the color changes of the electrolyte were noticed after the test. This is a good indication that the CCM is highly stable in the tested acidic environment and has no loss of gold nanoparticles.

Therefore, CCM loaded with Au NPs exhibited good catalytic activity, as well as excellent stability, as evidenced by the very low degradation coefficient from the stability tests. It showed a low onset potential value of 50 mV and a Tafel slope of 38 mV/dec, which is a better performance than the onset potential and Tafel slope values reported in the literature for chemically synthesized Au NPs, whose HER activity was tested under similar acidic conditions [19,45]. Thanks to the Au NPs that have been successfully transferred to the ionomer membrane in their nascent state, which retained their high active surface area, a competitive catalyst for HER applications is obtained. The potential reasons behind the higher electrocatalytic performance can be ascribed to the fact that the proposed direct transfer route has successfully embedded the Au NPs in their nascent state in the ionomer membrane with a strengthened chemical interaction that even enlarged the electrochemical active surface of the Au and as well as enhanced the proton transport properties of the catalyst-loaded ionomer membrane.

## 4. Conclusions

In conclusion, we have successfully prepared the nanosized catalyst gold particles (Au NPs) by RF magnetron sputtering onto liquid PEG, a versatile dry synthesis method. Our analysis, using STEM, AFM, and UV–vis spectroscopy, confirmed the nucleation of the spherically shaped Au NPs and duly characterized the size of the sputtered nanoparticles in the colloidal suspensions. To retain the nascent monodispersed state of the Au NPs for exploiting their electrocatalytic properties, a simple yet innovative technique has been developed to transfer them to an ionomer membrane. The resulting CCM was characterized using FE-SEM and AFM, confirming that monodispersed Au NPs are homogeneously embedded in the ionomer membrane. Moreover, the optimized transfer process completely removed the trapped PEG that enabled a stronger surface chemical interaction of the Au NP with the ionomer, as confirmed through XPS analysis. Finally, the optimized CCM loaded with Au NPs was tested for HER water electrolysis and compared with benchmark Pt/C. The catalytic results revealed that the CCM exhibited excellent HER performance, showing an outstanding onset potential of 50 mV and a Tafel slope of 38 mV/dec, owing to the full potential of the nascent gold nanoparticles that have the highest active surface area. Overall, this innovative work offers two premium strategies to produce ultrapure catalyst Au NPs by sputtering in liquids and facilitating their direct transfer to the ionomer membrane for water electrolysis applications.

## Figures and Tables

**Table 1:** Onset potential and Tafel slopes of the LSVs measured.

Sample	LSV Test	Onset Potential (V vs. RHE)	Tafel Slope (mV/dec)
Au NPs	Pre-Activation	−0.29	17.7 ± 0.3
	Post-Activation	−0.05	38.6 ± 0.4
	Post-CP	−0.05	38.1 ± 0.2
Pt/C	Post-Activation	−0.02	30.0 ± 0.5
	Post-CP	−0.02	23.6 ± 0.3

## Data Availability

The data presented in this study are available upon request from the corresponding author.

## Abbreviations

The following abbreviations are used in this manuscript:

CCM	Catalysts Coated Membrane
HER	Hydrogen Evolution Reaction
Au NPs	Gold Nanoparticles
PEG	Polyethylene glycol
B.E.	Binding Energy
PEMWE	Proton Exchange Membrane Water Electrolyzer
